# The complete mitochondrial genome sequence of *Halichoeres nigrescens* (Labriformes: Labridae)

**DOI:** 10.1080/23802359.2018.1511856

**Published:** 2018-10-08

**Authors:** Wei Shi, Shixi Chen, Hui Yu

**Affiliations:** a College of Life Science, Foshan University, Foshan, Guangdong, China;; b CAS Key Laboratory of Tropical Marine Bio-resources and Ecology, South China Sea Institute of Oceanology Chinese Academy of Sciences, Guangzhou, China

**Keywords:** Labriformes, *Halichoeres nigrescens*, phylogenetic relationship

## Abstract

The complete mitochondrial genome of marine fish *Halichoeres nigrescens* was sequenced by the high-throughput sequencing method. The genome is 17,252 bp in length, consisting of 13 protein-coding genes, 22 tRNA genes, two rRNA genes and one large non-coding region. The gene arrangement of *Halichoeres nigrescens* is identical to that of common fishes. Phylogenetic tree based on 13 protein-coding genes shows that *Halichoeres nigrescens* has a closer phylogenetic relationship to *Macropharyngodon negrosensis* than to *Halichoeres hartzfeldii*.

*Halichoeres nigrescens* (Bloch and Schneider, 1801), common name Bubblefin Wrasse, has pointed snout, 4 to 5 broad and dark brown bars on both side of body, one dark spot on the middle of anterior half of dorsal fin, and another spot at the base of pectoral fin. This fish inhabits in shallow weedy areas of rocky shorelines with little coral growth (Lieske and Myers 1994) and small individuals of this species have a habit to clean other fishes as previous records (Sadovy and Cornish 2000). *H*. *nigrescens* had been classified into family Labridae, order Perciformes in previous studies, but now family Labridae belong to order Labriformes (Nelson et al. 2016).

In this study, we first reported the complete mitochondrial genome of *H*. *nigrescens*, and analysed its phylogenetic relationship with some other species from family Labridae with one species of family Odacidae as outgroup, based on samples collected from Naozhou Island in Zhanjiang, China (geographic coordinate: N 20°53′20.11″, E 112°28′46.2″). The whole body specimen was preserved in ethanol and registered to the Marine Biodiversity Collection of South China Sea, Chinese Academy of Sciences, under the voucher number SW20181071709.

The complete mitochondrial genome of *H*. *nigrescens* is 17,252 bp in length (GenBank accession No. MH678616), including 13 protein-coding genes, two rRNA genes, 22 tRNA genes, one O_L_ (origin of replication on the light-strand) and one D-Loop (control region). The O_L_ is 53 bp in length, located in the cluster of five tRNA genes (*WANCY* region) between *tRNA*-*Asn* and *tRNA*-*Cys*. The D-loop is 1330 bp in length, located between *tRNA*-*Pro* and *tRNA*-*Phe*. Gene arrangement of this genome is identical to that of other common fishes, and most genes in this genome are encoded by the heavy strand (H-strand), except for *ND6* and eight tRNA genes (Boore 1999; Wang et al. 2017; Gong et al. 2017). Overall base composition values for the mitochondrial genome are 29.2%, 25.9%, 16.3%, and 28.5% for A, C, G, and T, respectively.

The phylogenetic relationships of *H*. *nigrescens* with 12 closely related species and one out group were analysed in this study. Complete mitochondrial genes of these 14 species are available on GenBank. The maximum-likelihood evolutionary tree (ML tree) was constructed by MEGA 7 (Kumar et al. 2016) based on 1st and 2nd codon sequences of 13 protein-coding genes.

In the ML phylogenetic tree, *H*. *nigrescens*, *Macropharyngodon negrosensis*, *Halichoeres hartzfeldii*, *Parajulis poecilepterus*, *Pteragogus flagellifer* and *Pseudolabrus eoethinus* formed a clade with a strong support. But *H*. *nigrescens* not clustered with *H. hartzfeldii*, whereas with *M. negrosensis* in this clade. Another clade was formed by *Cheilinus fasciatus*, *Calotomus japonicus*, *Bolbometopon muricatum*, *Scarus forsteni* and *Chlorurus sordidus.* The other clade was formed by *Choerodon schoenleinii* and *Bodianus oxycephalus.* These three clades mentioned above were all classified into family Labridae of order Labriformes (Figure 1). All above results show that *H*. *nigrescens* has a closer phylogenetic relationship to *M*. *negrosensis* than to *H*. *hartzfeldii*.

**Figure 1.**
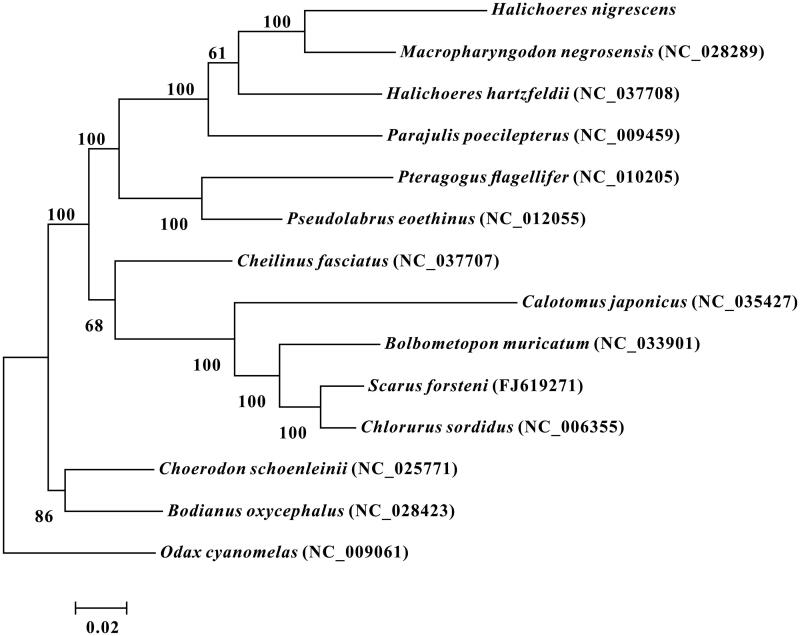
Maximum-likelihood phylogenetic tree was constructed based on 1st and 2nd codon sequences of 13 protein-coding genes of 14 species.
